# There was no call for immediate implementation of “Tetris” in clinical practice: Response to the commentary by Halvorsen et al. (2024)

**DOI:** 10.1038/s41380-024-02766-4

**Published:** 2024-10-04

**Authors:** Camille Deforges, Yvonnick Noël, Susan Ayers, Emily A. Holmes, Vania Sandoz, Valérie Avignon, David Desseauve, Julie Bourdin, Manuella Epiney, Antje Horsch

**Affiliations:** 1 Institute of Higher Education and Research in Healthcare, University of Lausanne, Lausanne, Vaud Switzerland; 2 Department of Psychology, Rennes 2 University, Rennes, France; 3 Centre for Maternal and Child Health Research, City, University of London, London, UK; 4 Department of Women’s and Children’s Health, Uppsala University, Uppsala, Sweden; 5 Department Woman-Mother-Child, Lausanne University Hospital, Lausanne, Vaud Switzerland; 6 Department of Obstetrics, Grenoble Alpes University Hospital, Grenoble, France; 7 Institute of Pedagogy and Applied Research, Limésy, France; 8 Department of Woman, Child and Teenager, Geneva University Hospitals, Geneva, Switzerland

**Keywords:** Psychiatric disorders, Psychology

We thank Halvorsen and colleagues for their commentary [1] on the Swiss TrAumatic biRth Trial (START) [2], a multicenter, double-blind, randomized controlled trial (RCT) with an active control group. START tested the efficacy of a single-session intervention, carried out within six hours following an unplanned cesarean section, to prevent maternal symptoms of childbirth-related posttraumatic stress disorder (CB-PTSD). It was a next step in our research program, in which a previous proof-of-principle RCT showed that women receiving this intervention had fewer intrusive childbirth-related memories during the first postpartum week than those receiving routine care [3]. At the end of our publication [2], we concluded that the next step would be an implementation study. However, in stark contrast to what Halvorsen et al. claim, this was not a call for immediate implementation of the intervention in clinical practice. Halvorsen et al.’s title claim is thus incorrect and misleading.

Halvorsen et al. rightly note that we did not operationalize the primary outcomes, as preregistered, by analyzing means, but rather by counting the symptoms present (scored ≥2, in accordance with validated ratings [4, 5]). Primary outcomes were group differences in the presence and severity of maternal CB-PTSD symptoms at six weeks postpartum on PCL-5^1^ and CAPS-5^2^ subscale and total scores. These primary outcome measures remain the same in the trial pre-registration (NCT03576586), study protocol [6] and the paper [2]. The way we analyzed them as symptoms counts is detailed in the paper, and we agree we should also have explicitly written this differed from the pre-registration. There was a strong statistical argument for using this different type of analysis. We analyzed PCL-5 and CAPS-5 (ordinal scales) using dedicated categorical models from Item Response Theory (IRT) [7]. Among IRT models, only Rasch models, when validated using suitable fit statistics [8], result in a global measure of severity from nominal presence of symptoms, or an ordinal assessment of their severity [e.g., 9, 10], that is reducible to a summed score. In START, routine IRT analyses showed that none of these models had an acceptable fit to the data (the RMSEA fit statistic ranged from 0.07 to 0.0943), and that a zero-inflation effect was present in the score data. The first result disqualified classical summed scores as a valid participant descriptor, while the second implied the rejection of means and standard deviations as valid group summaries, hence the deviation from the pre-registration. We therefore switched to symptom counts as a more appropriate measure of presence and severity of CB-PTSD symptoms. It would have been misleading to use invalid indicators, simply because they had been preregistered. Furthermore, we agree with Halvorsen et al. on the value of the recent CONSORT Outcome Extension [11], but it was not published at the time we finalized our manuscript. Halvorsen et al. incorrectly state that we did not respond to their request for information, but we replied to them well before we saw their commentary and our offer to meet went unanswered.

Halvorsen et al. describe our primary outcome results as “non-significant” and criticize our conclusion that the intervention had been beneficial. However, they seem to have misunderstood how a statistical approach based on Akaike Information Criterion (AIC) [12] allows to conclude in favor of an intervention effect. For each outcome variable, presented as a symptom count, we needed to infer i) a proper distributional model, ii) the presence of a potential zero-inflation effect, and iii) the existence of a group effect - either on the zero or non-zero part of the distribution. Given that distribution comparisons cannot be obtained from standard tests, we used a model comparison approach using information criteria, allowing us to test for all three aspects in a unique decisional procedure. The inferential decision criterion was the AIC: an intervention effect is statistically validated if the inclusion of the group variable, be it on the zero-inflation or non-zero part of the model, translated into a *diminished* AIC^3^. But while a reduced AIC indicates an intervention effect, it does not indicate the direction of the effect (i.e., if beneficial or negative). In the second stage, we therefore examined the sign of each coefficient individually (a negative *β* corresponds to *reduced* symptoms). While we reported their *p*-values, this was not necessary: as long as the AIC is lower, one can conclude a group effect. Note that, we chose to only comment on those effects for which *both* a lower AIC *and* a significant effect *p*-value were found, which constitutes an unusually conservative approach, contrary to what Halvorsen et al. suggest.

Thus, based on AIC and model coefficients, we reiterate that the intervention reduced the number of self-reported symptoms of CB-PTSD at both six weeks (total number of symptoms; intrusions and arousal subscales but not avoidance or negative alteration in cognitions and mood subscales) and six months postpartum (total number of symptoms; negative alterations in cognition and mood scale, as well as arousal subscales but not intrusions and avoidance subscales).

Halvorsen et al. expressed concern over missing outcome cases. Moving to symptom counts requires discarding participants with missing responses for fair between-participants and between-group comparisons. We note that >95% of cases were complete (only 6/128 cases at six weeks, and 5/113 at six months were discarded) as indicated in the paper [2]. Halvorsen et al. also wished for more information on participants who dropped out. This would indeed have been useful, but our ethical approval did not allow us to contact participants who dropped out. Nevertheless, the drop-out rate of 7.5% between randomization and primary outcome is well below the expected 20% [6].

Our paper sought to report results transparently, including null findings. For example, we stated that null findings on CAPS-5 and the intrusion diary were contrary to expectations (p.3847). We refrained from drawing mechanistic conclusions, which this type of clinical trial does not allow. In the discussion, we twice urged caution against overinterpreting results, e.g., “*the absence of group differences in clinical interviews warrants caution in interpreting the effects of the intervention*” and “*our results cautiously confirm its efficacy in the secondary prevention of CB-PTSD symptom development*”.

We are pleased Halvorsen et al. acknowledge the strengths of this study: adequate randomization and efforts to maintain blinding. START was designed with a multidisciplinary steering committee of international experts, who oversaw data collection and approved the independent statisticians’ advice to conduct primary outcome analyses by symptom count (7/6/2022). The intervention effect was assessed using both self- and clinician-reported validated measures up to six months. START had regular oversight by an independent trial monitor. Analyses were conducted by an independent statistician blinded to group allocation. The sample size was calculated based on the effect sizes found in our previous RCT [3], with power calculations carried out by an independent statistician and approved by the ethics committee^4^.

In summary, we sought to conduct START with care and rigor from planning to publication. Participants who received the intervention developed fewer self-reported CB-PTSD symptoms than those in the active control group, for up to six months post-intervention, in accordance with AIC statistics. This pattern of results constitutes a meaningful step forward, given the unmet need for interventions for mothers after traumatic childbirth and that the intervention is acceptable, requiring few resources. We readily acknowledge that START has several limitations, as highlighted [2], but we do not see that these justify the criticism of being “*actively misleading*”. Halvorsen et al.s’ claim that we made a “*premature call for implementation of Tetris in clinical practice*” is incorrect, since nowhere in our paper did we call for the intervention to be immediately used in clinical practice. Rather, we wrote that “*Future research may thus evaluate its implementation*“. Implementation research is a form of research by definition done before actual clinical practice.

## Footnotes

1 PTSD Checklist for DSM-5

2 Clinician-Administered PTSD Scale for DSM-5

3 Importantly, as a measure of the expected distance (in the Kullback-Leibler sense) to the true structure of the data [12], the computation of this distance for one model does not impact the same computation for another model on the same data. Thus, multiplicity corrections are inappropriate here.

4 It is well-known from simulation studies [13] that applying sample size calculations based on the Gaussian distribution to non-symmetric count distributions, leads to conservative results, inflating the required sample size by as much as 50%, depending on the effect size. To take but one example, replicating on the total PCL-5 symptom count at six months of the simulation by Cundill and Alexander [13], and using the AIC, a total sample size of *N* = 86 participants would have been sufficient to detect an average difference of one unit count (from 1.55 to 2.55, which we observe), in the presence of overdispersion (we observe a dispersion parameter of *k* = 0.53), with a power of 0.80.
